# Earthquake rebuilding and response to COVID-19 in Nepal, a country nestled in multiple crises

**DOI:** 10.7189/jogh.10.020367

**Published:** 2020-12

**Authors:** Bipin Adhikari, Akihiko Ozaki, Sujan Babu Marahatta, Komal Raj Rijal, Shiva Raj Mishra

**Affiliations:** 1 Centre for Tropical Medicine and Global Health, Nuffield Department of Medicine, University of Oxford, Oxford, UK; 2 Mahidol-Oxford Tropical Medicine Research Unit, Bangkok, Thailand; 3 Department of Breast Surgery, Jyoban Hospital of Tokiwa Foundation, Iwaki, Fukushima, Japan; 4 Manmohan Memorial Institute of Health Sciences, Kathmandu, Nepal; 5 Nepal Open University, Lalitpur, Nepal; 6 Central Department of Microbiology, Tribhuvan University, Kathmandu, Nepal; 7 Nepal Development Society, Chitwan, Nepal

COVID-19 pandemic has affected the health, social and economic integrity of low- and middle- income countries (LMICs) in an unprecedented way [1]. Income losses are projected to exceed $220 billion in these countries where 55% of the global population lives. As high as 75% of the population in LMICs simply lack the access to soap and water for minimal hygiene which is now more critical for COVID-19 prevention [2]. In Nepal, 18 752 cases of COVID-19 were reported until 27th July 2020, however, the impacts are far beyond these numbers because of the cumulative impacts due to COVID-19, lock down and the damages due to earthquakes (**Figure 1**). In 2015, Nepal’s two earthquakes claimed 9000 lives and left 23 000 injured, and surprisingly profound and sustained impacts on health system persisted even after five years of earthquakes [3,4]. Moreover, mobility restriction against COVID-19 has put a tremendous pressure on poor and vulnerable population who struggle with the daily wage for food in Nepal. In this piece, we attempt to explore long-term impact of Gorkha earthquakes in the communities threatened by COVID-19 pandemic and vice versa. We specifically explored how the long-term impacts due to the earthquakes are compounded by the evolving current pandemic.

**Figure 1:**
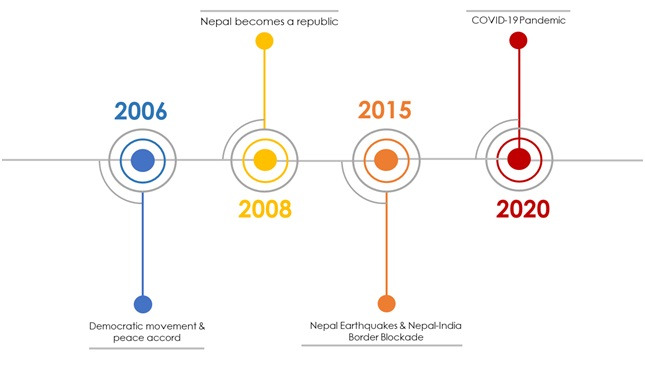
Nepal’s two decades of timeline coming to COVID-19 pandemic. Nepal signed a peace accord with a Maoist rebellion that lasted for 10 years in 2006. With a new political wave, Nepal abolished monarchs from power. In 2015, Nepal was hard hit by two devastating earthquakes, and India’s economic blockade. While rebuilding process was sluggish and tepid even after five years, Nepal shares the pandemic burden due to COVID-19.

## IMPACTS OF GORKHA EARTHQUAKES AND COVID-19 PANDEMIC ON NEPAL’S HEALTH SYSTEM

Nepal’s health system suffers from poor coverage and quality of health services particularly at the existing ~ 5000 health facilities [4,5]. Nepal’s already strained health system was worsened by the damages due to the Gorkha earthquakes; and is inevitably going to be impaired by the current pandemic [6]. The constraints in human and material resources to fight against the current pandemic have already echoed into public health care sectors; and without private sector’s engagement it is likely to exhaust the health system’s capacity [7]. Indeed, the future recovery and response efforts against COVID-19 are likely to encounter similar challenges as in 2015 earthquakes. More importantly, Nepal’s health system and its functioning is enervated by corruption, and has deterred Nepal’s earthquakes rebuilding and COVID-19 response [8].

COVID-19 pandemic mirrors the impact of Gorkha earthquakes, with impacts more prominent on vulnerable population living in rural and border areas that includes wage-based labourers returning from India or the stranded population desperately seeking government’s attention. Mortality associated to Gorkha earthquakes was high among vulnerable population that included children <10 years, adults >55 years, females, and from remote mountains [5]. The higher risk of infections and mortality among these specific population shows important bottlenecks in Nepal’s health system and its ability to plan and prepare for future disasters/pandemics.

Nepal’s ~ 1600 km porous border with India allows high importation of infectious diseases. For instance, half of the malaria cases in Nepal is imported by returning labour migrants from India [9]. However, the potential scale of cross-border transmission was largely overlooked during the COVID-19 response. Porous nature of the border, and lack of screening and quarantine allowed high infiltration across the border. It thus appears that repeated failure to learn from the past disasters (economic blockade following the earthquakes) and the disease epidemiology (high importation of malaria, dengue, including HIV) have transcended to the current public health measures against COVID-19. Devolving and implementing the testing capacity and resources at provincial, municipal and community level, and more importantly, private sector’s engagement is critical to mitigate the current crisis.

**Figure:**
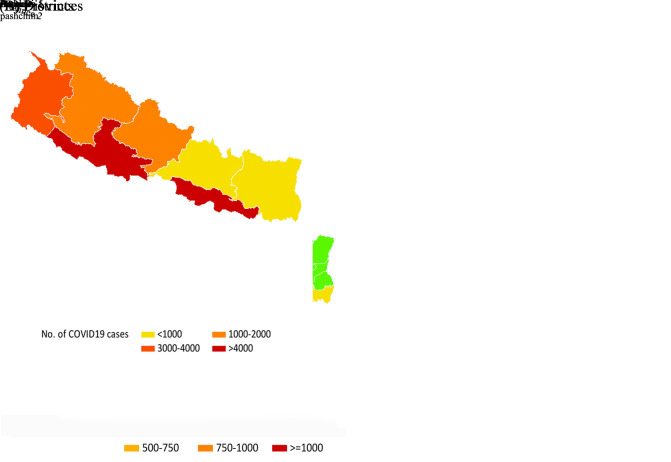
Photo: COVID-19 cases in Nepal. At the province level (**A**) and districts level (**B**) in Nepal. This visualization includes 18 752 cases of COVID19 cases reported until 27 July, 2020. Geospatial map was created using GMAP procedure in SAS 9.4. The shape files were obtained from the Government of Nepal, Ministry of Federal Affairs and Local Development and were publicly available for unrestricted use (https://data.humdata.org/dataset/admin-shapefiles-of-nepal-mofald).

### Relationship between earthquakes, and COVID-19

The Gorkha earthquakes had a pervasive impact on health system and planetary health [3], and its implications on the current COVID-19 pandemic are compelling. Infrastructural damages (500 000 houses) [4], the social and economic consequences due to Gorkha earthquakes further triggered 500 000 to emigrate for jobs and opportunities [3], to an extent that extreme poverty in few villages led young males to sell their kidneys [10]. Poverty led migration is a hard reality in the lowest quartile of the population who live in less than a dollar a day [3]. Amidst existing challenges to livelihood, COVID-19 pandemic will escalate the vulnerability and poverty. For instance, the mobility restriction (particularly across the border) against COVID-19 stranded a mass exodus of labour migrants in poor conditions, and the government could not reach the optimal capacity in testing them [11].

In addition, the chronicity due to high unemployment, corruption and precarious economy (dependent on remittance) is further weakened by current pandemic and is likely to expulse more emigrants from rural to urban and Nepal to outside [12]. Rural to urban migration has been exerting enormous pressure on urban management, green space, water and sanitation in Nepal [12,13]. The poor living conditions in urban areas (crowded living, poor hygiene) are a great risk to an explosion of COVID-19 as it also can compound the other infectious diseases.

### Impacts due to COVID-19 pandemic

COVID-19 pandemic has already strained Nepal’s economy, with projected drop in the GDP growth rate from 7.1 to 4% [14]. Remittance constitute more than a fourth of Nepal’s GDP and its estimated drop due to COVID-19 will severely prostrate Nepal’s economy. Despite these looming challenges, government’s response to the COVID-19 crisis has been cumbersome; corruption charges in procurement of testing kits have added serious threats over the public health preparedness against COVID-19 [15]. In absence of effective testing, tracking and tracing strategy, lockdown has been the only option, which however, can have plethoric ramifications in the country’s economy, health and the infrastructure with patent impacts on rebuilding; with hopes for restoration of ravaged heritage sites pushed further away. Acutely, current drop in remittance—a major source of Nepal’s economy is likely to have chronic consequences for years to come and may reduce and shift the budget allocated for earthquake rebuilding.

Apart from the immediate health impacts due to COVID-19, it is likely to affect the long-term health of the population. Specifically, the lockdown ceased the food chain and its production with impacts in overall agricultural productivity and food security [16]. These impacts can persist beyond the current pandemic aggravating the undernutrition, hunger, and mental health and it is likely to hit hard the poor households in Nepal, echoing with other LMICs.

## CONCLUSION

At five years post Gorkha earthquakes, the country’s rebuilding process has been nestled in the current COVID-19 crisis. Many systemic health challenges have emerged impacting population that needs attention. Immediate measures are required to support the health system including strategies to curb the COVID-19 pandemic and maintain the rebuilding for future.
